# A qualitative content analysis of the experience of hypospadias care: The importance of owning your own narrative

**DOI:** 10.3389/fped.2023.1118586

**Published:** 2023-02-17

**Authors:** Lottie Phillips, Nicklas Dennermalm, Lisa Örtqvist, Hedvig Engberg, Gundela Holmdahl, Magdalena Fossum, Anders Möller, Agneta Nordenskjöld

**Affiliations:** ^1^ Department of Women’s and Children’s Health and Center of Molecular Medicine, Karolinska Institutet, Stockholm, Sweden; ^2^ Department of Social Work, Stockholm University, Stockholm, Sweden; ^3^ Department of Paediatric Surgery, Astrid Lindgren Children's Hospital, Karolinska University Hospital, Stockholm, Sweden; ^4^ Department of Gynecology and Reproductive Medicine, Karolinska University Hospital, Stockholm, Sweden; ^5^ Department of Paediatric Surgery and Faculty of Health and Medical Sciences, Rigshospitalet, University Hospital of Copenhagen, Copenhagen, Denmark; ^6^ School of Public Health and Community Medicine, Institute of Medicine, University of Gothenburg, Gothenburg, Sweden

**Keywords:** hypospadias, patient perspective, disorders of sexual development (DSD), in-depth interview, qualitative content analysis

## Abstract

**Objectives:**

There is a lack of studies on men's individual experiences of living with hypospadias. We aimed to explore the personal experiences of having hypospadias in relation to healthcare and surgery.

**Subjects and methods:**

Purposive sampling was used to include men (aged 18 and over) with hypospadias representing different phenotypes (from distal to proximal) and ages in order to maximise the variation and richness of our data. Seventeen informants, aged 20–49, were included in the study. In-depth semi-structured interviews were conducted between 2019 and 2021. Inductive qualitative content analysis was used to analyse the data.

**Results:**

We identified three categories: (1) *Having surgery*, which comprised the decision to operate, the experience of having surgery, and the outcomes of surgery; (2) *Going to the doctor*, which focused on follow-up care, re-entering care in adolescence or adulthood, and the experience of healthcare interactions; (3) *Being informed*, both about hypospadias in general, as well as about your specific body and medical history. There was overall a large variation in experiences. The latent theme across the data was the importance of *owning your own narrative*.

**Conclusion:**

The experience of men with hypospadias in healthcare is complex and varied, highlighting the difficulty of fully standardised care. Based on our results, we suggest that follow-up should be offered in adolescence, and that ways of accessing care for late onset complications be made clear. We further suggest clearer consideration for the psychological and sexual aspects of hypospadias. Consent and integrity in all aspects and all ages of hypospadias care should be adapted to the maturity of the individual. Access to trustworthy information is key, both directly from educated healthcare staff and if possible, from websites or patient-led forums. Healthcare can play a key role in providing the growing individual with tools to understand and address concerns that may develop relating to their hypospadias through life, giving them ownership over their own narrative.

## 1. Introduction

Hypospadias results from a disruption in the foetal development of the penis and urethra, leading to a urethral opening proximal to the tip of the glans, varying on a spectrum from very proximal (perineal) to very distal (glanular). Typically associated with a cleaved foreskin, boys can have smaller than average penile length. Many boys also have some degree of penile curvature (1, 2). While the reported prevalence varies across countries, in Sweden it has increased over time to the current 1 in 125 (3, 4). The aetiology of hypospadias includes genetic and environmental risk factors, often related to foetal androgen function (1).

For centuries the treatment for hypospadias has been surgical, with techniques improving significantly over time (5). Almost everyone diagnosed with hypospadias in Sweden is treated surgically in infancy or early childhood. Outcomes are generally very good, while the risk of complications increases significantly in proximal cases from circa 5%–15% to 50% in some studies (6). Late complications as well as other problems related to hypospadias can become apparent years after surgery, for instance during puberty (6). Despite this, following hypospadias surgery, most patients are not followed past childhood or adolescence (7).

While research has started to emerge exploring the experiences of parents and healthcare professionals in hypospadias care, there is yet no research looking more in-depth at the experience of the patients themselves (8–10). One Swedish follow-up study using questionaries found that most men with hypospadias did not feel they needed more medical or psychological support, but that men with proximal hypospadias were more likely to say they wished for more care (11). Most of the general literature on the experience of children and adolescents with chronic or congenital medical conditions focus on illnesses such as diabetes mellitus and cystic fibrosis, which entail much more contact with healthcare and more need for active treatments than most boys living with hypospadias, limiting the scope for comparison, although some experiences may be shared (12). Given the sensitive nature of having a genital variation, the experience of hypospadias treatment and care could be hypothesised to elicit issues relating to shame or devaluation as shown in qualitative studies investigating the experiences of individuals with other disorders of sexual development (13, 14).

Given the limited knowledge provided by previous studies of the patient´s experiences relating to hypospadias care, we aimed to further explore those experiences using inductive qualitative methods. Ultimately, we aimed to generate knowledge that could be used to improve healthcare practices for boys and men with hypospadias.

## 2. Materials and methods

This study is presented in accordance with the Standards for Reporting Qualitative Research (SRQR) guidelines (15). A qualitative paradigm was selected given the very sensitive and personal subject matter about which very little is known. We make the philosophical assumption of interpretivism, meaning that reality is subjective and socially constructed. Inductive qualitative content analysis was selected as it would help us to address our research question without any prior hypothesis or theory. We defined and assessed the trustworthiness of our study by considering credibility, dependability, transferability, and confirmability (16, 17).

### 2.1. Study setting

In Sweden, hypospadias is most often detected shortly after birth and all cases are referred to specialist clinics. The patients are examined and may undergo further tests, for instance to assess urinary flow, or genetic or hormonal testing in familial, very proximal, or complex cases. Surgery is currently recommended for patients with hypospadias in infancy, usually around 1 year of age (7). However, in the final decades of the 20th century in Sweden, surgery was most often done before starting school at around age 4–6. Hypospadias is now primarily treated in a day surgery setting, but most cases were admitted to hospital for several nights during the study period. Follow-up typically continues to a maximum age of 15, with varying frequency and focus depending on phenotype and whether there are complications but often including genital exams and uroflowmetry. If paediatric urology patients need continued care, they must be referred to other specialists.

### 2.2. Sampling

Inclusion criteria were diagnosis of hypospadias and/or related conditions [i.e., penile curvature or small penis (less than 2.5 SD from the mean)], a minimum age of 18, and fluency in English or Swedish. Exclusion criteria were, for ethical reasons, ongoing severe physical or mental comorbidity. Purposive sampling was used to assure variation in age and phenotype of hypospadias. Specifically, this was done through our clinic, personal knowledge, a post on social media, snowball sampling (i.e., one informant recruits another) as well as self-referral (further details in Supplementary Material, Section S1).

### 2.3. Data collection

In-depth, semi-structured interviews were used for data collection. An interview guide was developed by the researchers based on topics that are relevant from clinical experience, subject-specific knowledge, and previous research (2, 11). We started by asking the informant to speak freely about their experience living with hypospadias. Then followed the topics of childhood and up-bringing, healthcare, self and identity, interpersonal relationships, and fatherhood (full translated interview guide in Supplementary Material, Section S3). The 17 interviews took place between May 2019 to August 2021 and lasted around 40 min (range: 14–68 min). Interviews were recorded in full and transcribed verbatim by the first author. Transcripts were checked for accuracy by the second author (further details in Supplementary Materials, Section S2). The emerging data was discussed at intervals throughout the interview process. This continued until the two interviewers agreed that sufficient data saturation was achieved, meaning that no significant new information (in variation or theme) was added, and then no further informants were recruited.

### 2.4 Data analysis

**Figure 1:**
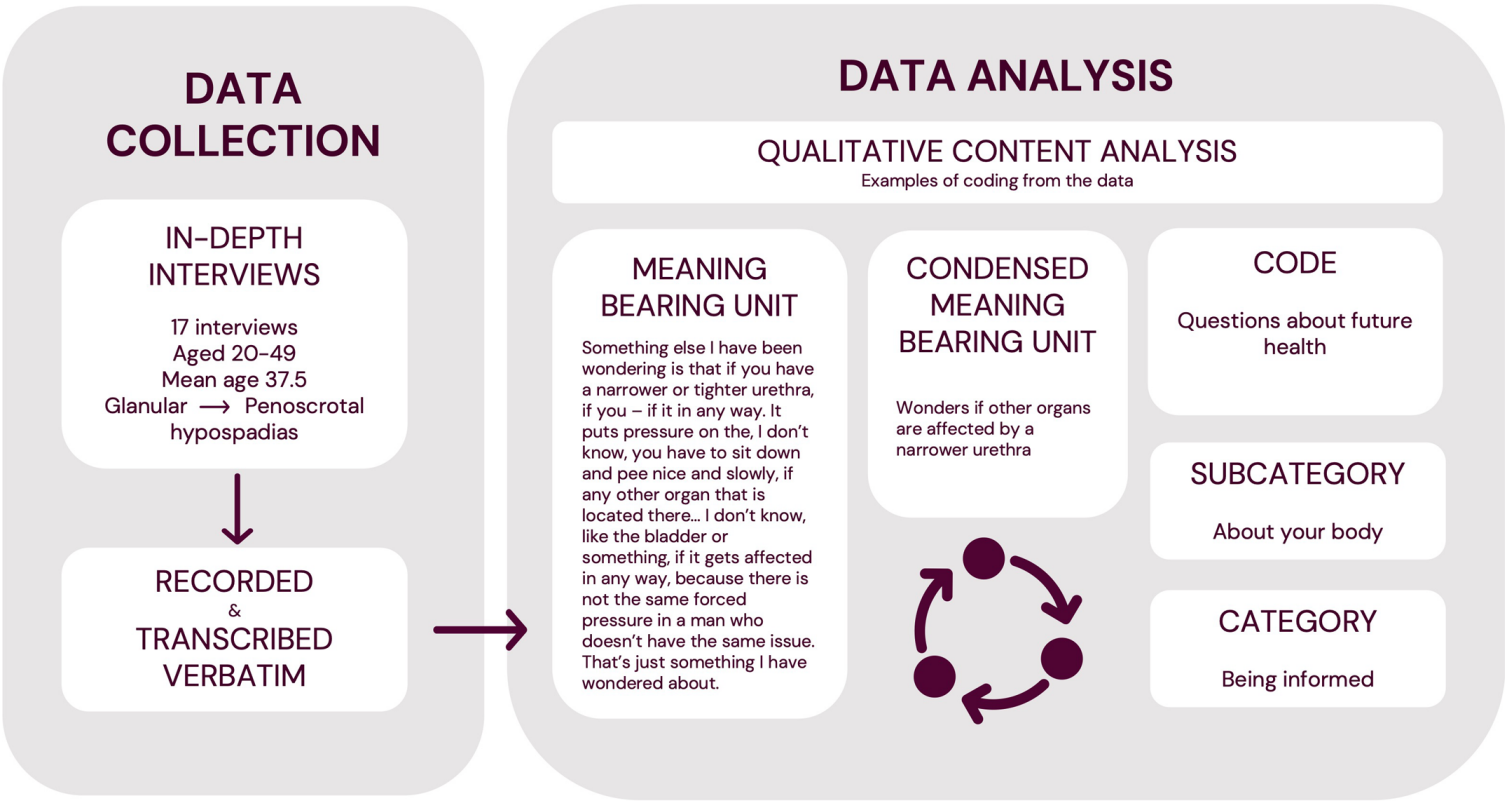
Overview of the data collection and analytical process.

Once all interviews were completed, inductive content analysis was performed following the approach of Graneheim and Lundman (17). Nvivo (Release 1.7) was used to facilitate data handling. The analysis was conducted by the first author, who is bilingual in Swedish and English. Firstly, all interviews were read through several times. Meaning bearing units were identified, condensed, sorted, and abstracted into codes which were grouped into subcategories and categories. The process was inductive meaning that no codes, subcategories, or categories were formed prior to analysis based on previous theoretical frameworks. The analysis was also iterative, meaning that as it developed, each level of abstraction was re-assessed and regrouped several times with input from the other authors to reach a full and meaningful interpretation of the underlying data. Finally, any latent themes were discussed and identified by assessing whether any recurring, underlying meaning was found across different categories (Figure 1).

### 2.5 Ethics

The Swedish Ethical Review Authority had approved the study and all participants were included after informed written and oral consent. Transcripts were assigned codes which were linked *via* a two-step coding system to any personal information. Any identifiers mentioned during the interview were blanked in the transcripts. Sounds files are kept securely at the university for a minimum of 10 years, only accessible to the core researchers. All participants were given the opportunity to raise questions relating to hypospadias with an expert and those that needed further medical or psychological support were assisted in the process.

## 3. Results

A total of 17 informants were included (Figure 1). They had a wide range of phenotypes from small penis (less than 2.5 SD from the mean) and/or penoscrotal hypospadias, to glanular hypospadias, as well as varied socioeconomic and social backgrounds. As well as having hypospadias themselves, a few informants had sons who also had hypospadias.

**Figure 2:**
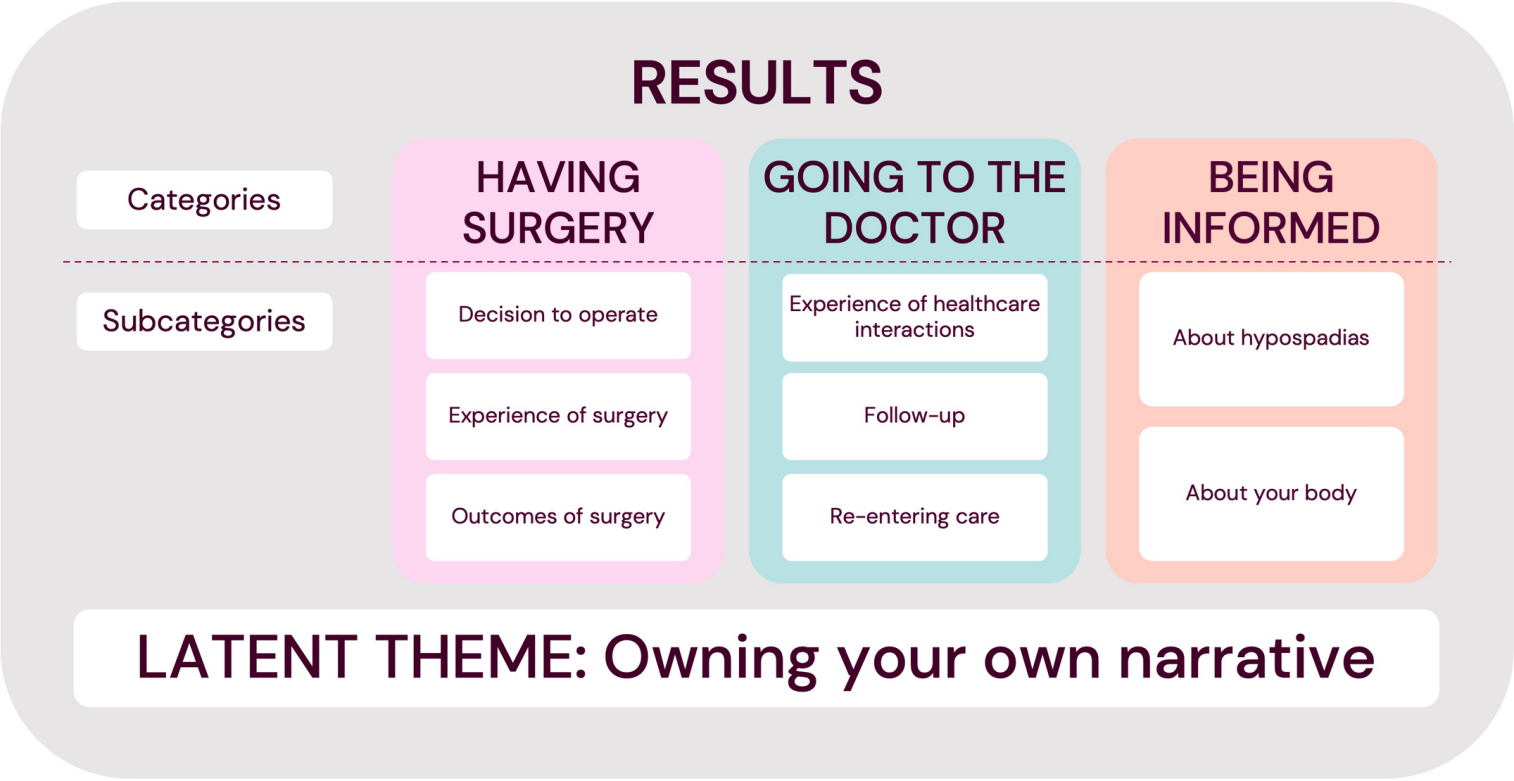
Overview of the manifest categories and latent theme.

Through analysing the 17 in-depth interviews, three manifest categories were identified relating to being a patient: “***Having surgery”***, “***Going to the doctor”*** and “***Being informed”***. We also identified a latent theme of the importance of owning your own narrative (Figure 2).

### 3.1. Having surgery

#### 3.1.1. Decision to operate

Almost all informants had had surgery during childhood and, in general, this meant that the decision was beyond their control. Instead, their parents or doctors had chosen to operate. Some viewed that choice as very positive: “*It was a bit weird, peeing [like that]. Psychologically. It was hard to see it, so that’s why they made the decision to do it. So, it was a good decision” (IN6)*. Others viewed surgery in early childhood as neutral, or problematic: “*I think it could be good to have a debate about, at least these distal or whatever you call it, the milder forms of hypospadias, that maybe they should be done when the child can give their consent. Or when they are a little bit more intellectually developed, and cognitively, so they can make a conscious decision. It feels like abuse almost, and like something that’s been taken from you.” (IN9)*. One informant whose son had hypospadias decided to postpone surgery to let the son decide for himself, but later regretted it: “*He feels that we should have made the decision when he was a baby. (…) And now – I have admitted that, so has his mother, that with hindsight, we should probably have done it when he was a baby. But then, at the time, it felt like it’s his body, and then he should get to make that decision” (IN3)*.

With regards to the reasons to operate, informants described understanding that the first surgery or surgeries had been done primarily to lengthen, widen or reconstruct the urethra. Some informants named psychological discomfort about how you pee or how you look as the primary motive for surgery. A distinction was made between cultural and medical motives for surgery: “*I was asking questions and then it was like why did you chose to have me circumcised like this? And then it was ‘no, but it wasn’t a cultural or religious decision, you needed to have surgery because you have a short uretha’– well she didn’t say it was a short urethra, she said ‘you peed in every direction’” (IN4)*. The concept of distinguishing between what was considered cosmetic or medical was also raised, with differing ideas about what constituted “*cosmetic*”, ranging from for instance the appearance of the foreskin to penile curvature.

For surgeries later in life, informants felt more involved in the decision-making process but stated the importance of clear communication about the scope of the surgery and the risks and benefits, to make an informed decision: *“I could pee better after the first surgery than after the second surgery. So, it was not an improvement. That has a lot to do with me [not] understanding what he was going to do and him not communicating well enough, and so on. (…) [The surgeons] spend so little time talking to me and then he cut me up for life you know, so it’s a big deal, it should be talked about a little bit more. Maybe have some tests, I don’t know. Document things, they didn’t document anything.” (IN15)*. Some informants faced with deciding to have more surgeries as adults felt that it was not worth risking something that was not “*that bad”*, for instance if it was viewed as “*cosmetic*”. Some had already moved on with their lives while they would have been more interested in further surgeries if they had been younger. Others viewed surgeries as key to achieving satisfactory sexual function, urination, or overall well-being, for example by correcting penile curvature or previous complications, and improving aesthetic outcomes.

#### 3.1.2. Experience of surgery

Memories from surgeries in childhood were fragmented and incomplete, and the overall experience ranged from traumatic to neutral, or even positive. It could also be boring, as having to lie down a lot, and lie still, was frustrating as a child. Watching TV was one positive memory, as well as eating ice lollies: “*[I remember] that someone rolled in a TV, so you got to watch an animated film and play some video game, because we didn’t have that at home. So, I think I thought it was pretty fun (…). I don’t remember it as anything difficult or scary” (IN8)*. Specific memories of more traumatic experiences included being put under general anaesthesia, having a catheter, having an enema, and being in physical pain. Memories of being scared were described, especially when not understanding what was happening, or being physically restrained: “*I don’t really remember anyone actually talking to me and like explaining- and I was 6 years old, I think. But no one explained or said, ‘this is going to happen’, instead- I remember that I was very scared. And I cried when I was being put under general anaesthetic. And I remember that they, the staff, tried to comfort me. For me, it was scary, because I didn’t understand what they were going to do or what was going to happen” (IN3)*. Feeling alone could also make you nervous, although informants generally remembered at least one parent being present, while some described being visited by other family members as well, and even receiving gifts.

For those operated after starting school, being admitted to hospital and being away from school or home made you feel different to your peers. Age at time of surgery could also have a general impact on the experience, and surgeries closer to puberty could be especially sensitive, compared to surgeries in early childhood. One informant contrasted feeling more unsettled by surgery as a teenager: “*It was pretty sensitive as well; I mean because it’s a sensitive age. (…) But then, I think it was a little- it was a little more uncomfortable that- the fact that it’s so intimate and a bit unsettling that someone has like cut that particular organ. To wake up and look and there was some bandage but there was like blood, and it actually hurt quite a lot” (IN8)*.

#### 3.1.3. Outcomes of surgery

The informants found it harder to judge the outcome of surgeries in early childhood, having to relate it more to a general perception of their own appearance and function. In contrast, the outcome of surgeries later in life could be compared in terms of before and after, ranging from a large improvement to no difference at all or a significant step in the wrong direction. Some informants struggled with their disappointment and trying to accept that their problems might not get any better. Others focused on things being as good as they could or were very satisfied. Expectations related to how “*bad*” the starting point was perceived to be: “*I think that everything has worked well and considering that I- my experience is that I’ve had pretty major surgeries. There are a lot of different things I’ve had to have surgery for. And that it’s still ended up being this good in the end” (IN10)*.

When discussing specific outcomes, informants described having a urethral opening varying from the very tip to further down the glans. Some described it as abnormally wide or downward facing. The other most noticeable outcome was being circumcised. The aesthetic outcome was also discussed, and while some informants were pleased, others felt that the aesthetics could have been considered more during surgery: “*Of course, it would have- I mean when they did the procedure anyway, they might as well have closed the foreskin too. Of course, that’s what I would have wanted. It actually felt pretty primitive, what they did to me- my experience is pretty much that they cut a bit to widen the opening a little, but there was no aesthetic consideration at all” (IN3)*. Some informants specifically raised penile size and were concerned that surgery had impacted penile length, or that something could have been done to increase the length surgically or pharmaceutically, but that it was now too late.

Surgical complications described by informants included urological issues such as strictures or the formation of urethral “*pockets*”, persistent curvature, urinary tract infections, hair growth in the urethra, and pain. Some had developed significant difficulties urinating, with a slow stream or problems fully emptying their bladder, while others described never having any issues. Those who had needed to use intermittent self-dilation in adulthood had found it painful, uncomfortable, re-traumatising, or even eventually impossible to do, and hoped for a more long-term solution: “*Has anyone like done something wrong or something, then maybe it could be fixed? (…) I just wish that someone would put me to sleep and fix this stricture so that I can pee and be normal” (IN5)*. Loss of sexual sensation for those who experienced it was hypothesised by informants to relate to different causes such as circumcision, but also that erectile tissues had been removed, or that important nerves had been damaged.

Outcomes in general were not described as static, with some informants finding it improved over time, while others experienced that the new urethral opening gradually separated, tore, or widened, or they developed problems urinating well into their thirties. It could also take time after surgery to heal and grow more secure in the results. Fear that something would tear, or break, could impact daily life, including sexual activities.

### 3.2. Going to the doctor

#### 3.2.1. Experience of healthcare interactions

Overall, there was a large variation in experiences of healthcare interactions from only positive to strongly negative.

One factor the informants highlighted was the degree of awareness given that the patient is a child, and the vulnerability that entails. It was raised as important that the doctor speak directly to the child as a “*whole human-being”* and respect the child's autonomy in a way that is appropriate to the level of maturity: “*They didn’t really speak to me but to my parents. They spoke over my head, like adults often do when children are involved” (IN11).* Informants hypothesized and hoped that this had changed over time and that healthcare staff are more understanding towards children than they were a few decades ago. One informant contrasted his own experience to the experience of accompanying his son to the doctor: *“When I think about how my son was treated, I think that they could have explained things better to me and– yeah, explain the procedure. More information and more empathy, I would say” (IN3)*.

Genital exams were described as a sensitive experience, especially in conjunction with a painful or difficult procedure. Getting an involuntary erection during a genital exam could be particularly embarrassing. Informants felt that professional, supportive, and calm staff could help make the experience less uncomfortable. In contrast, receiving negative reactions or comments from healthcare staff about for instance penile appearance could be very upsetting. It was advised that parents and siblings be asked to leave before intimate exams if the child is a bit older or uncomfortable having them there. The presence of students was also mentioned, and having lots of people come and go, being examined multiple times, or being shown as an “*example*”, especially without consent, could be felt as a violation of integrity. Having new doctors was similarly viewed as potentially problematic, while having continuity could build trust and comfort.

More extreme examples of treatment in interactions that lacked empathy, or any autonomy, were described as traumatic and could lead to an avoidance of doctors and healthcare staff that lasted for years: “*I thought he was so hardhanded, that doctor (…). It took many, many, many years before I dared to go to a doctor again after this. Put plainly, I developed a phobia of doctors” (IN1)*. Other informants described contrasting interactions with nurses, urotherapists, and doctors who were patient, empathetic and professional: “*I was going to see that nice doctor, that I went to see. I don’t remember the name, but yeah– ‘we’re going to the doctor there’ and then I remember it being like a little thing between me and my mum, that we thought he was pretty nice, so it was a fun thing” (IN13)*.

#### 3.2.2. Follow-up

Depending on the age of the informant, the severity of the hypospadias, and other factors, there was variation in the extent of follow-up from not recalling having had any at all to viewing regular visits as an obvious part of childhood.

If follow-up was remembered, it was generally experienced to mainly, or solely, focus on peeing: “*I remember many times, sitting in that chair and drinking lots before and then peeing. I don’t really know what they measured, but yeah, I remember that” (IN10)*. In other cases, the purpose and focus of visits was unclear. Talking was raised as important, while the skill of doctors to address sensitive or psychological issues was generally described as lacking: “*Today when you asked me how I am, it was the first time that a medical professional had asked me that related to my hypospadias. Never before has psychological well-being been brought up” (IN14, added in written correspondence after the interview and included in the analysis with consent)*. Focus on sexual function and sensitivity was also considered lacking, without available qualified support to help with both more psychosexual issues but also to discuss and optimize anatomical or physiological function, in relation to their background of hypospadias and related surgeries. Some informants wondered whether a lack of time limited the opportunity for more sensitive topics and questions.

A specific desire for more qualified psychological support was clearly raised, or at least the availability for those who need it due to for instance larger physical issues or a lack of support at home: “*If you could feel that (…) society wanted to support you in that, and not just view it as (…) sort of like with dental care, ‘now we will take care of all your dental care and then you’ll be an adult and you are on your own, so I hope you will have learnt enough’. But (…) it’s also not like you even get any dental hygiene recommendations either, I just get what I can physically, and then it’s left to the parents basically to handle any insecurity that comes up and then if it's not something that can be talked about– which can often be quite taboo, then you are pretty alone” (IN12)*.

Puberty was raised as an important period for both psychological and sexual health, and follow-up was suggested to extend into puberty, or if possible, even further into adolescence. While it was suggested that not all patients at that age would want, need, or be susceptible to support, and that it is very hard to say what is “*right”*, the opportunity to have support was viewed as important: “*I think it would have been- maybe not strictly necessary for me, but I can imagine that for others it would have been good to have a follow-up visit at the beginning of puberty and during puberty. I think so. Because I think it can be stigmatizing for some. To be different, to be circumcised. (…) I have thought about that, through life, that it probably would have been good” (IN17).* Informants suggested that a follow-visit around that time would allow both for an exchange of information, but also to identify any healthcare needs such as surgical complications.

While some felt their parents were very supportive, others described them as having a lot of practical control over follow-up visits, even in puberty, which could take a sense of agency away from the patient. Concern was raised that some parents might have cancelled visits without them knowing, and one informant described experiencing his father cancelling his last follow-up visit: “*And then it was at the 15-year follow-up visit, when mum felt that it was dad who should take the responsibility, but he didn’t want to go so it didn’t happen. I haven’t felt that I’ve been able to talk to mum about it, and she probably just saw it more as dad’s responsibility as a- it was like a male thing. But he just felt ‘it’s ok, it’s good’. So (…) I’m disappointed with him, that he [cancelled the visit]” (IN9)*.

Regardless of the extent of follow-up, it always eventually ended when the informants grew too old for paediatric urology and no transitional care was offered. This could be quite disturbing for those who had had regular visits and did not understand why they ended: “*It was so important, to go to [the doctor], but then that was it. It was unpleasant, going to the follow-up visits, but if it had continued for maybe another 2, 3 years or something, until I was 18, that would have felt safer for me, you know? But why it didn’t continue, I think that’s a bit weird, I don’t know” (IN1)*. Others felt that they were done: “*I feel like it was finished then, somehow (…), I was pretty content I guess, that they had tried to fix what they could, and it actually turned out pretty well” (IN8)*.

#### 3.2.3. Re-entering care

Some of our informants had sought help after leaving paediatric urology, due to complications or remaining issues or concerns with either function or aesthetics.

When there was a need for re-entering care, the informants highlighted the importance of knowing how to access care, but equally of knowing what kind of problems could arise. Not understanding that a certain symptom or issue could be related to hypospadias, or not knowing that there is a potential treatment option, could lead to postponing seeking care for years or indefinitely: “*I’m glad that I did something about it, the last surgery (…), because [that] means that I feel a lot better today. So, it helps. I think a lot of people probably avoid it as well, going back to healthcare, because- well, partly because you can’t imagine that maybe it could be better” (IN10)*. It was also not always apparent that there were complications. For instance, it was raised that decreased sensitivity or other issues with sexual function might not have been noticeable before having any sexual experiences.

Once informants had decided they wanted help, there were further potential barriers. One barrier was delaying contacting healthcare because of fear and avoidance. Others related to structural issues in the healthcare system. Informants described experiences of being referred over and over, having to repeat their issues or be re-examined, and a general lack of qualified doctors as well as a low level of knowledge about hypospadias amongst healthcare professionals in general: “*Primary care has been a little– the way they treat me is still lacking, I have to say. (…) For me as a patient, it’s not fun having to sit there and explain my whole life story before there are any decisions about what I really need help with, and there are examinations and it’s a whole long process every time” (IN11)*. In some cases, lack of time and resources had had an impact, both during a doctor's visit, and when waiting to get appointments or surgeries. While there was understanding that life-threatening conditions like cancer were prioritized, waiting could be physically and emotionally demanding.

After getting a chance to get help, some had very positive experiences, while others felt their issues were not taken seriously and they were just told that nothing was wrong. One informant discussed seeking help abroad: “*There are like two surgeons in the [entire country] that do adult hypospadias. And based on past knowledge I just don’t think they- like the plastic surgeon that I had, he is good as a plastic surgeon, I think, but I don’t think he understands the condition as well as like the Doctor XX in the USA” (IN15)*. The impact of losing access to a trusted doctor or a way in could be significant.

### 3.3. Being informed

#### 3.3.1. About hypospadias

There was great variation in knowledge and understanding about what hypospadias is or means, what causes it, and whether it is a malformation or “*disease*”. In some cases, even the word hypospadias was unknown until adulthood. Of particular importance was knowing how common hypospadias is, as informants felt that knowing that would make it less stigmatizing: *“I think it would have helped a lot of people (…), to tell them it’s not that rare (…). Then I think it would take the edge off being– that you are different. Because that’s what children absolutely don’t want to be, no matter what” (IN17)*.

Based on the information the informants had received growing up, focus was put on having a short urethra. During the interviews, aspects such as not having a foreskin, penile size, or issues peeing were thought by some informants to not be at all connected to hypospadias. Others discussed reaching an understanding that hypospadias existed across a spectrum, and that it could be severe and even mean being born with unclear biological sex or having impaired hormone function. One informant described feeling that their condition was more complex: “*It’s a developmental disorder that’s bigger than just the urethra being in the wrong place, it is. I have– I was a weak child, physically. And a small penis” (IN12)*.

Those who were more knowledgeable had generally searched for information themselves. While the internet had some answers it was pointed out that websites or forums that exist for other conditions were lacking for hypospadias. There was a wish to know more about what happens to men with hypospadias, both in terms of physical health and more psychosocial well-being, and some informants felt there should be spaces and ways for boys and men with hypospadias to interact. Informants also mentioned they were glad that this study was being conducted to find out more: “*It would have been a bit interesting to know, [how] others have been– that’s why I think this study is good too, to find out how people are later on in life with hypospadias” (IN10)*.

#### 3.3.2. About your body

As well as understanding hypospadias more generally, informants spoke about being informed, or indeed lacking information, about their own bodies and medical history. The informants understanding of their surgical history varied from detailed to being uncertain about what type of surgery had been performed, why or even whether they had had surgery at all. Some informants did not find out that they were born with hypospadias until years later. One informant spoke about finding out that he had hypospadias as an adult, and whose responsibility it was to inform him: “*I can feel it’s sad that it wasn’t talked about in the family (…). [I have] more laughed at my parents, that they weren’t good enough to bring it up, but it also made me think about like healthcare. Of course, I partly blame– or mostly blame– my parents, but I think that if I hadn’t received the letter from you, I wouldn’t have known” (IN4)*.

While some felt they had the information they needed, others had been very affected by not understanding different symptoms or aspects of their anatomy: “*I have always wondered (…). Why is it like this (…)? It’s just been stuck in my mind, like what is it? It’s stupid that I waited until I was [this] age before I asked anyone, but now at least I’ve asked someone and it feels like you will point me in the right direction which feels great” (IN5, talking about surgical complications)*. Some wanted to know more about their prognosis or had concerns about their future health.

Questions could have been unclearly answered, not have an answer, or else informants had never been given the opportunity to ask questions at all. To find out more, some had looked at old medical records but had remaining concerns about what things meant or found conflicting information: “*So, I have always been told that they didn’t know the sex but that’s not true either, really, when I look at the medical records. There is a lot, when I got hold of the medical records and got more information, there is soooo much that doesn’t add up with what my parents told me” (IN1)*. Although some informants did not feel personally affected, they generally advised that healthcare provide more written and oral information, especially given that so much from childhood, both what had been done and said, had been forgotten, leaving them knowing less about their own bodies than their parents or doctors.

### 3.4. Latent theme: owning your own narrative

Across the categories, a recurring theme was the importance of having ownership over your own narrative. This encompassed aspects such as informed consent, bodily autonomy, and knowing and understanding your own past, present, and future. Lacking information, words, or the security and tools necessary to discuss your body and your needs could negatively impact everything from health-seeking behaviour and medical decision-making to general satisfaction and well-being. Owning your own narrative could also encompass not wanting or needing more information or support but having the power to choose.

## 4. Discussion

Quantitative methods are often used to study for instance quality of life or experience of care. However, surveys and similar methods cannot give an in-depth understanding of personal experience, which is why we aimed to analyse the personal experience of hypospadias care using qualitative in-depth interviews with men born with hypospadias. We recruited informants from a broad perspective concerning severity of hypospadias, surgical history, and sociodemographic factors to cover as many aspects as possible. Our results highlight the importance of information, both about hypospadias in general and specifically about the history, care, and prognosis of the individual, as well as patient-centred and empathetic treatment which allows for active and open discussions in an age-appropriate manner. Availability to access healthcare in adolescence was widely requested, as well as clearer ways to get qualified care throughout life, and broader sexual and psychological support. Surgery could be seen as positive and necessary, although it was also highlighted that it cannot be undone and may impact long-term functional and aesthetic outcomes. While many raised the importance of phenotype, other factors such as surgical outcomes, the presence or lack of other support systems, and unanswered questions also dictated the need for care.

Deciding to operate naturally falls on the parents and surgeons in early childhood, and recent studies have investigated the dynamics and complexities of that shared decision-making process, as well as ways to improve it (18–20). As the patient grows however, issues of informed consent should gradually be transferred to the patient themselves. Active involvement of the child in paediatric care decisions as far as possible has many benefits including increased satisfaction and preparation for transition into adult care (21). Our data highlights that being informed and involved in an age-appropriate manner throughout childhood can improve the overall experience and sense of autonomy, which has also shown to be important in previous qualitative studies on children with chronic illnesses (12). We have chosen not to quantify how many informants were satisfied or dissatisfied with having had surgery in early childhood, as this could lead to incorrect assumptions about men with hypospadias in general. The large variation we found highlights the difficulty in trying to find a “one-size-fits-all” solution.

Some of our informants discussed the concept of cosmetic surgery, and not prioritising or choosing to do something that was viewed as “*cosmetic*”, while their definition of cosmetic overlapped with issues that affected function or caused pain. This supports the editorial previously published by Snodgrass and Bush presenting a concern that classing something as cosmetic could lead to delaying or avoiding potentially beneficial treatment, and instead recommending the terms “reconstructive” and “aesthetic” (22).

Outcomes described by our informants were consistent with previous literature and clinical experience (23). Some of our informants described that they had developed strictures and other issues well into adulthood, in concordance with some previous studies on long-term surgical outcomes (24, 25). While some of our informants were concerned that functional complications had arisen from tissue such as corpus spongiosum being removed, it is important to highlight that modern hypospadias surgery is conservative reconstructive surgery. However, certain surgical procedures used to treat penile curvature may increase the risk of nerve damage or erectile dysfunction, although improved surgical techniques have reduced the risk (24).

Our informants placed different focus on specific aspects of their surgical results, which were broadly grouped as urination, aesthetics, and sexual function. Severe voiding dysfunction for those who experienced it could greatly impact everyday life. In contrast, if urination was less affected, outcomes related to appearance and sexual function became central, while the experience was that healthcare still primarily focused on voiding. Less focus being placed on both sexual and psychological outcomes is mirrored in many of the available patient reported outcome (PRO) tools and scales currently used, as well the focus of the most cited articles in the field of hypospadiology (26, 27). In developing hypospadias specific PROs and health-related quality of life scales, all these domains (i.e., urination, aesthetics, sexual, and psychological) should be considered. To help understand and judge especially aesthetic and sexual outcomes, some informants suggested more objective documentation and tests before and after surgery.

Empathy, integrity, and professionalism in healthcare were clearly important in our data. Looking at the age of our participants, it appears that the younger ones had generally more favourable experiences, indicating improvements over time, although this should be interpreted with caution.

Our results show the great importance of taking into consideration the growing and changing needs and vulnerabilities of the paediatric hypospadias patients. This supports a consensus statement based on a qualitative survey of healthcare professionals that raised child-oriented professionalism as one of the primary domains in caring for children born with ambiguous sex (28). Some informants shared that traumatic healthcare experiences during childhood that could lead to extensive and long-lived avoidance of medical care. In contrast, Duarsa et al. found that none of their patients, that had an operation, showed signs of clinical post-traumatic stress disorder (PTSD) on standardised questionnaires in early childhood (29). To avoid experiences of trauma, we suggest that the specific examples raised by our informants be considered, such as of lack of control and information, especially in painful and vulnerable scenarios.

Our informants specifically raised the importance of calm and caring medical staff during intimate exams and procedures. Correspondingly, Engberg et al. found that women with congenital adrenal hyperplasia experienced that repeated genital exams were both traumatic and unnecessary, but that professional staff could significantly improve the experience (13). Our results also highlighted how having parents or siblings present could make the experience even more uncomfortable. However, performing genital exams or invasive procedures without any guardian or chaperone present could potentially also raise issues or make the patient more uncomfortable. Morgan et al. found that while the views of adolescents on medical chaperones varied, many, especially female, participants appreciated being asked and able to choose (30). To respect the integrity and autonomy of the individual, we suggest that more consideration be focused on the wishes of the child in these scenarios.

Having to constantly see new doctors was contrasted by our informants to the sense of security in having the same doctor over time. Similarly, Nah et al. found that youths with Hirschsprung or anorectal disease had positive experiences of healthcare interactions only after an extended time period getting to know and trust their providers (31). More generally, continuity of care in paediatrics has been shown to have several benefits, including increased child and parent satisfaction (32). While most boys with hypospadias have a small number of clinic visits, continuity as far as possible could help to improve the experience and may increase the chance to identify or anticipate needs.

Although the physical changes during puberty have been raised as a motive for follow-up of hypospadias patients during those years, an international specialist survey showed that only around half of all hypospadias surgeons followed their patients to puberty, and only 27% into adulthood (7). Örtqvist et al. found that as many as 45.5% of men with proximal hypospadias wished for more medical follow-up, and some wished for more psychological support (11). Our results have further elucidated that psychological support, especially in adolescence, could help the individual to deal with concerns or insecurities relating to stigma or identity. In addition, the need for competent urological support not only encompassed urination but also aesthetics and sexual function. Transitional care should be considered if remaining issues are identified during puberty. The European Academy of Paediatric consensus statement highlights key components for successful transition, including preparation and collaboration (33). As mentioned in our results, not everyone with hypospadias may want or need follow-up in adolescence. Being given the option of clear information and support, however, may be more broadly adapted. Even those with distal hypospadias may struggle with unanswered questions at least during some periods of life, while neutral and correct information should generally not introduce concerns. We cannot speak specifically to whether it is always advisable to reach out to very mild cases who have had no, or minimal, surgical treatment.

When re-entering care as adults, both patient's delay and delay in the healthcare system were described. Helping boys and men with hypospadias to normalise and grow safe and comfortable with hypospadias care, avoiding traumatising or stigmatising experiences, and providing them with the information necessary to identify healthcare needs may help reduce patient's delay. Lack of knowledge amongst professionals and having to start all over again every time you seek medical help, were raised both by our informants and in a systematic review of qualitative literature on living with a rare disorder, even though hypospadias is not actually rare (34). While ensuring that doctors across general practice and urology have sufficient knowledge of hypospadias is one potential path for improvement, perhaps closer to hand would be ensuring that accessible and clear routines and guidelines are in place for the referral, or self-referral, to a smaller number of trained, competent, and interested professionals.

Just as among medical professionals, public knowledge and awareness about hypospadias is poor. Snodgrass et al. found that 86% of 260 parents surveyed had not heard of hypospadias prior to their son's diagnosis (35). Many of the knowledge gaps that our informants raised about hypospadias were mirrored in studies by Chan et al. on parents of boys with hypospadias, such as questions about possible long-term outcomes and not understanding different anatomical features (8, 10). The importance of information was also highlighted in a qualitative study interviewing women with androgen insensitivity syndrome, who felt that information was often withheld from them and wanted more transparency in healthcare, similarly to some of our informants (14).

The internet, and in particular social media, had been shown to be used by a large majority of parents and patients to find health-related information and guidance, but also to have a lack of correct information about hypospadias that can be understood by non-expert parties (8, 36, 37). By providing trustworthy and accessible information across different forms of platforms, experts in the hypospadias field could help combat both the lack of information and the spread of misinformation. A study by Jacobs et al. surveying primarily parents but also patients with other congenital anomalies found that 84% wished for healthcare professionals to actively participate in their Facebook group (38).

### 4.1. Trustworthiness, strengths, and weaknesses

The primary strength of our study is the unique and rich in-depth data on the personal experience of hypospadias care, as told by adult men born with hypospadias. We recruited men of varying ages, phenotypes, and demographic backgrounds to try to capture as far as possible the variation in experience and increase dependability. All data relevant to the study aim was included in analysis, which was performed by the first author. Quotes and clear descriptions and examples of the analytical process are included to help the reader to assess credibility. The co-authors of this study have a variation of both formal and informal education, and this broad scope of our research group contributed nuance and depth to our understanding of the data, allowing for discussions on contrasting perspectives. These discussions about the interpretation of the data and the formation of the results were used to judge and improve confirmability.

In contrast to quantitative design, sample size is not related to statistical analyses. Instead, for each individual study, factors such as the aim, the richness of the data, and the type of analysis will impact data saturation. Our sample size is reasonable in relation to previous empirical and theoretical literature, and our individual interviews provided large amounts of informative data (39, 40). This allowed us to analyse and present both variation and depth in experience, with themes that kept recurring when we ended our data collection. While our results can only fully reflect the experiences of the 17 men included in this study, these factors indicate that experiences of hypospadias care in our study setting were more broadly encompassed by our findings.

Though there is variation amongst our informants, including different religious and cultural backgrounds, all our informants experienced healthcare in Nordic countries. While hypospadias treatment with regards to types of surgery and the general structure of follow-up share many similarities in different countries, there will be cultural and practical differences across healthcare systems. The experiences voiced in this study also give a partly historical perspective on hypospadias care in our setting. We have attempted to provide significant information about our study setting, through background information and descriptions by informants, to allow the reader to judge transferability to their specific context.

### 4.2. Implications, conclusions, and future directions

**Figure 3:**
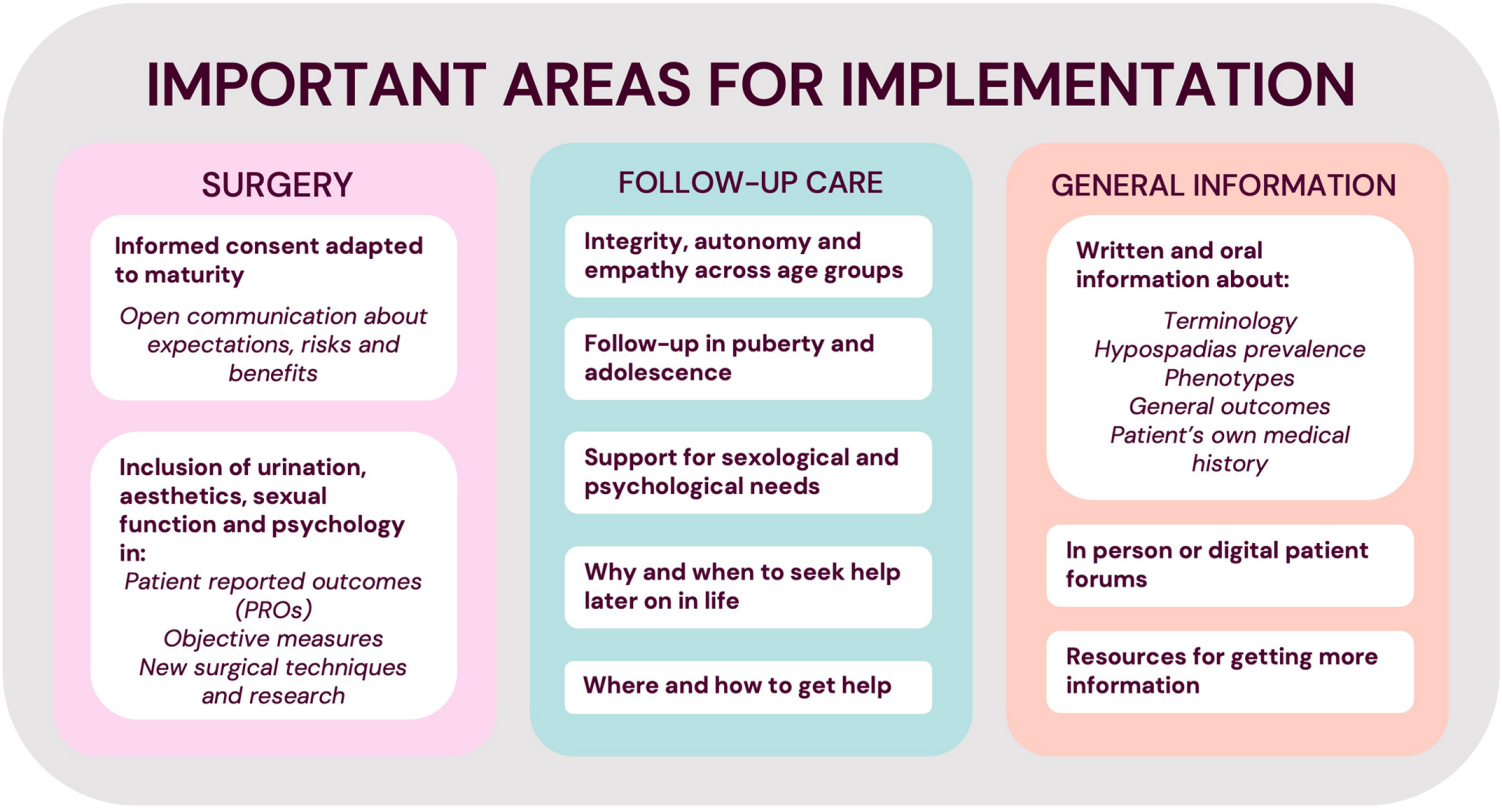
Summary of important factors to consider in hypospadias care.

**Figure 4:**
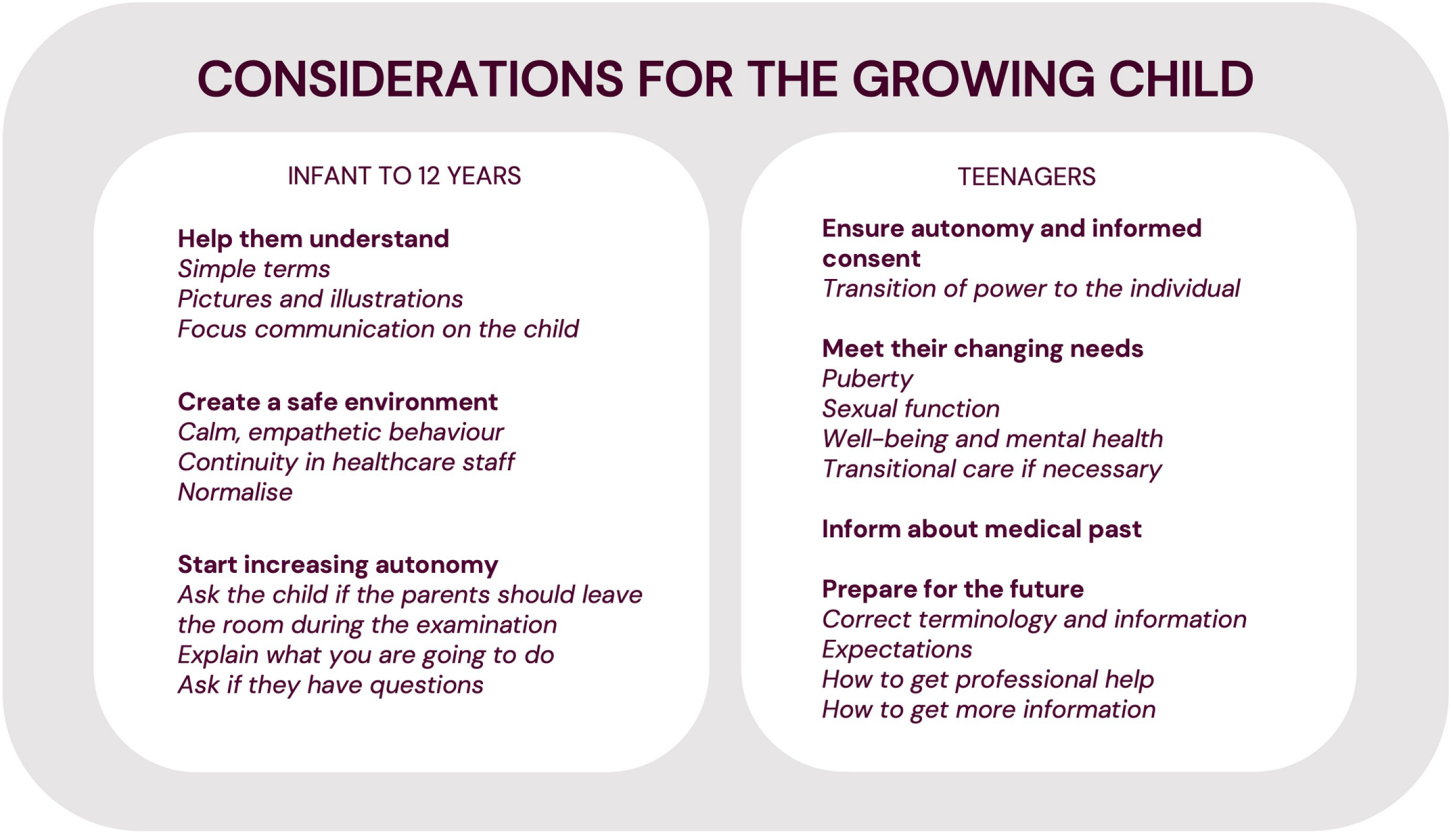
Summary of recommended considerations for the growing child in hypospadias care.

This is the first qualitative study on the personal experience of hypospadias care. It highlights the importance of owning your own narrative, and the results have many practical implications. Briefly, we recommend clear and easy access to correct information about hypospadias, as well as to specialist care throughout life, including other professions if necessary, such as psychologists, sexologists or andrologists. The existence of interested and competent healthcare professionals for adolescent and adult care is essential, with systems for self-referral if possible. We have summarised our overall suggestions for implementation in Figure 3. Suggestions for considering the changing needs of the growing child in hypospadias care are summarised in Figure 4. More specific suggestions raised by some of our informants are listed in our Supplementary Materials (Section S4).

For further exploring experiences of healthcare and treatment of boys and men with hypospadias, in-depth interviews or focus groups may be used to address more specific topics or to evaluate for instance scoring systems or care protocols prior to implementation. While it remains important to investigate the perspectives of parents and healthcare staff, we wish to emphasise the importance of involving men, and if possible, boys, with hypospadias themselves to reach insights that may not be readily shared in a traditional patient-carer setting and give a voice to those most directly impacted.

## Data Availability

The datasets presented in this article are not readily available because of ethical and legal restrictions due to the personal nature of the in-depth interviews. Requests to access the datasets should be directed to lottie.phillips@ki.se.

## References

[1] van der Zanden LFM van Rooij IALM Feitz WFJJ Franke B Knoers NVAM Roeleveld N. Aetiology of hypospadias: a systematic review of genes and environment. Hum Reprod Update. (2012) 18(3):260–83. 10.1093/humupd/dms002 22371315

[2] Örtqvist L Fossum M Andersson M Nordenström A Frisén L Holmdahl G Long-term followup of men born with hypospadias: urological and cosmetic results. J Urol. (2015) 193(3):975–82. 10.1016/j.juro.2014.09.103 25268894

[3] Skarin Nordenvall A Frisén L Nordenström A Lichtenstein P Nordenskjöld A. Population based nationwide study of hypospadias in Sweden, 1973 to 2009: incidence and risk factors. J Urol. (2014) 191(3):783–9. 10.1016/j.juro.2013.09.058 24096117

[4] Yu X Nassar N Mastroiacovo P Canfield M Groisman B Bermejo-Sánchez E Hypospadias prevalence and trends in international birth defect surveillance systems, 1980–2010. Eur Urol. (2019) 76(4):482–90. 10.1016/j.eururo.2019.06.027 31300237 PMC7265200

[5] Hadidi AT. History of hypospadias: lost in translation. J Pediatr Surg. (2017) 52(2):211–7. 10.1016/j.jpedsurg.2016.11.004 27989535

[6] Long CJ Canning DA. Hypospadias: are we as good as we think when we correct proximal hypospadias? J Pediatr Urol. (2016) 12(4):196.e1–196.e5. 10.1016/j.jpurol.2016.05.002 27296789

[7] Steven L Cherian A Yankovic F Mathur A Kulkarni M Cuckow P. Current practice in paediatric hypospadias surgery; A specialist survey. J Pediatr Urol. (2013) 9(6):1126–30. 10.1016/j.jpurol.2013.04.008 23683539

[8] Chan KH Panoch J Carroll A Wiehe S Cain MP Frankel R. Knowledge gaps and information seeking by parents about hypospadias. J Pediatr Urol. (2020) 16(2):166.e1–166.e8. 10.1016/j.jpurol.2020.01.008 32061490 PMC8562056

[9] Roen K Hegarty P. Shaping parents, shaping penises: how medical teams frame parents’ decisions in response to hypospadias. Br J Health Psychol. (2018) 23(4):967–81. 10.1111/bjhp.12333 30054962

[10] Chan KH Panoch J Carroll A Wiehe S Downs S Cain MP Parental perspectives on decision-making about hypospadias surgery. J Pediatr Urol. (2019) 15(5):449.e1–449.e8. 10.1016/j.jpurol.2019.04.017 31383519 PMC6824977

[11] Örtqvist L Andersson M Strandqvist A Nordenström A Frisén L Holmdahl G Psychosocial outcome in adult men born with hypospadias. J Pediatr Urol. (2017) 13(1):79.e1–79.e7. 10.1016/j.jpurol.2016.08.008 28087231

[12] Shorey S Ng ED. The lived experiences of children and adolescents with non-communicable disease: a systematic review of qualitative studies. J Pediatr Nurs. (2020) 51:75–84. 10.1016/j.pedn.2019.12.013 31926405

[13] Engberg H Möller A Hagenfeldt K Nordenskjöld A Frisén L. The experience of women living with congenital adrenal hyperplasia: impact of the condition and the care given. Clin Endocrinol (Oxf). (2016) 85(1):21–8. 10.1111/cen.13054 26941069

[14] Alderson J Madill A Balen A. Fear of devaluation: understanding the experience of intersexed women with androgen insensitivity syndrome. Br J Health Psychol. (2004) 9(1):81–100. 10.1348/135910704322778740 15006203

[15] O’Brien BC Harris IB Beckman TJ Reed DA Cook DA. Standards for reporting qualitative research: a synthesis of recommendations. Acad Med. (2014) 89(9):1245–51. 10.1097/ACM.0000000000000388 24979285

[16] Elo S Kyngäs H. The qualitative content analysis process. J Adv Nurs. (2008) 62(1):107–15. 10.1111/j.1365-2648.2007.04569.x 18352969

[17] Graneheim UH Lundman B. Qualitative content analysis in nursing research: concepts, procedures and measures to achieve trustworthiness. Nurse Educ Today. (2004) 24(2):105–12. 10.1016/j.nedt.2003.10.001 14769454

[18] MacNevin W MacDonald A Hong P MacLellan DL Anderson PA Romao RLP. Shared decision-making for pediatric elective penile surgery. Can Urol Assoc J. (2022) 16(10):340–345. 10.5489/cuaj.7761 35621289 PMC9565063

[19] Chan KH Misseri R Cain MP Whittam B Szymanski K Kaefer M Provider perspectives on shared decision-making regarding hypospadias surgery. J Pediatr Urol. (2020) 16(3):307–15. 10.1016/j.jpurol.2020.03.015 32307325 PMC8562057

[20] Chan KH Misseri R Carroll A Frankel R Moore CM Cockrum B User-centered development of a hypospadias decision aid prototype. J Pediatr Urol. (2020) 16(5):684.e1–684.e9. 10.1016/j.jpurol.2020.07.047 32863127 PMC7686073

[21] Miller VA. Involving youth with a chronic illness in decision-making: highlighting the role of providers. Pediatrics. (2018) 142:S142–8. 10.1542/PEDS.2018-0516D 30385620 PMC6220652

[22] Snodgrass W Bush N. Is distal hypospadias repair mostly a cosmetic operation? J Pediatr Urol. (2018) 14(4):339–40. 10.1016/j.jpurol.2018.06.004 29970334

[23] van de Grift TC Rapp M Holmdahl G Duranteau L Nordenskjold A. Masculinizing surgery in disorders/differences of sex development: clinician-and participant-evaluated appearance and function. BJU Int. (2022) 129:394–405. 10.1111/bju.15369 33587786 PMC9292912

[24] Rague JT Cheng EY. Complications after hypospadias repair: are we adequately counseling families? J Urol. (2022) 208(3):528–529. 10.1097/JU.0000000000002828 35748130

[25] Snodgrass W Bush N. Do new complications develop during puberty after childhood hypospadias repair? J Urol. (2022) 208(3):696–701. 10.1097/JU.0000000000002738 35536670

[26] Bhatia VP Hilliard ME Austin PF Mittal AG. Evaluating quality of patient-reported outcome measures in patients with hypospadias. J Pediatr Urol. (2021) 17(1):50–8. 10.1016/j.jpurol.2020.11.043 33371965

[27] O’Kelly F Nason GJ McLoughlin LC Flood HD Thornhill JA. A comparative bibliometric analysis of the top 150 cited papers in hypospadiology (1945-2013). J Pediatr Urol. (2015) 11(2):85.e1–85.e11. 10.1016/j.jpurol.2014.11.022 25819379

[28] Streuli JC Köhler B Werner-Rosen K Mitchell C. DSD And professionalism from a multilateral view: supplementing the consensus statement on the basis of a qualitative survey. Adv Urol. (2012) 2012:185787. 10.1155/2012/185787 22829810 PMC3399384

[29] Duarsa GWK Pratiwi DA Tirtayasa PW Yudiana W Santosa KB Oka AAG Functional and cosmetic urethroplasty outcome, emotional stress after genital examination, post traumatic stress disorder, and ages at the time of urethroplasty as potential risk factor causing psychosocial disorder of hypospadia children. Open Access Maced J Med Sci. (2019) 7(9):1452–5. 10.3889/oamjms.2019.227 31198453 PMC6542407

[30] Morgan R Katzman DK Kaufman M Goldberg E Toulany A. Thanks for asking: adolescent attitudes and preferences regarding the use of chaperones during physical examinations. Paediatrics and Child Health (Canada). (2016) 21(4):191–5. 10.1093/PCH/21.4.191 PMC4934159 27429571

[31] Nah S Ong C Lie D Marimuttu V Hong J Te-Lu Y Understanding experiences of youth growing up with anorectal malformation or hirschsprung’s disease to inform transition care: a qualitative in-depth interview study. Eur J Pediatr Surg. (2018) 28:67–74. 10.1055/s-0037-1605351 28837998

[32] O’Malley AS. Current evidence on the impact of continuity of care. Curr Opin Pediatr. (2004) 16(6):693–9. 10.1097/01.mop.0000142488.67171.02 15548934

[33] Mazur A Dembinski L Schrier L Hadjipanayis A Michaud PA. European Academy of paediatric consensus statement on successful transition from paediatric to adult care for adolescents with chronic conditions. Acta Paediatr. (2017) 106(8):1354–7. 10.1111/APA.13901 28471516

[34] von der Lippe C Diesen PS Feragen KB. Living with a rare disorder: a systematic review of the qualitative literature. Mol Genet Genomic Med. (2017) 5(6):758–73. 10.1002/MGG3.315 29178638 PMC5702559

[35] Snodgrass P Snodgrass W Bush N. Parental concerns of boys with hypospadias. Res Rep Urol. (2021) 13:73–7. 10.2147/RRU.S285626 33604312 PMC7882430

[36] O’Sullivan NJ Nason G Manecksha RP O’Kelly F. The unintentional spread of misinformation on ‘TikTok’; A paediatric urological perspective. J Pediatr Urol. (2022) 18(3):371–5. 10.1016/j.jpurol.2022.03.001 35331640

[37] Salama A Panoch J Bandali E Carroll A Wiehe S Downs S Consulting “dr. YouTube”: an objective evaluation of hypospadias videos on a popular video-sharing website. J Pediatr Urol. (2020) 16(1):70.e1–70.e9. 10.1016/j.jpurol.2019.11.011 31928900 PMC7186156

[38] Jacobs R Boyd L Brennan K Sinha CK Giuliani S. The importance of social media for patients and families affected by congenital anomalies: a Facebook cross-sectional analysis and user survey. J Pediatr Surg. (2016) 51(11):1766–71. 10.1016/j.jpedsurg.2016.07.008 27522307

[39] Malterud K Siersma VD Guassora AD. Sample size in qualitative interview studies: guided by information power. Qual Health Res. (2016) 26(13):1753–60. 10.1177/1049732315617444 26613970

[40] Guest G Bunce A Johnson L. How many interviews are enough?: an experiment with data saturation and variability. Field Methods. (2006) 18(1):59–82. 10.1177/1525822X05279903

