# Supplementary material for: A qualitative content analysis of the experience of hypospadias care: The importance of owning your own narrative
Source: Front Pediatr. 2023 Feb 17;11:1118586. doi: 10.3389/fped.2023.1118586 (PMC9982152; doi:10.3389/fped.2023.1118586)
Supplement: Supplementary file 1 [file Datasheet1.docx]

Supplementary Material

# Details of the sampling process

While we hypothesized that primarily those with proximal hypospadias would be affected in any negative way, this study covers a new area, and we therefore ultimately chose to investigate experiences across the phenotype spectrum. Initial participants were identified from a previous clinical follow-up study conducted by our group and contacted via letter. ^1^ There was no follow-up communication or reminders for those who did not respond to the initial invitation, and they were not asked to inform us of why they did not wish to participate out of respect for their right to freely decline participation. In total, 68 people were contacted via letter, of which 9 were included in the study and 2 withdrew after initial approval prior to interview (no informants withdrew participation after being interviewed). Initially, only those who lived within reasonable travelling distance from Stockholm were contacted to allow for physical interviews. In the later stages of the study, due to COVID-19, all interviews were conducted via zoom which allowed for the participation of informants outside of Stockholm. Further informants were recruited through self-referral (they contacted us to ask about our research), through a clinic visit with a son who also had hypospadias, a post on the Facebook page of the Swedish Federation for Lesbian, Gay, Bisexual, Transgender and Queer Rights (RSFL) and snowball sampling (i.e., one informant recruits another). One of the informants was recruited through personal connection with one of the interviewers but was interviewed by the other interviewer. All participants lived in and had received hypospadias care in a Nordic country (Sweden or Norway). Different sampling methods were introduced at different points during the interview process to further maximise demographic variation, as well as to try to maximise the richness and depth of our data.

# Details of the interview process and interviewers

The interviews were mainly conducted in a one of two neutral meeting room environments at departments at the Karolinska Institute adjacent to the University Hospital, i.e., not in a clinical environment. One of the interviews was conducted in a clinic room at a paediatric surgery out-patient clinic. None of the informants were interviewed in the same environment that they had previously received healthcare in. No one was present other than the interviewer and informant. Some later interviews were conducted over zoom due to the covid-19 pandemic or geographical distances. One interview was conducted over the phone due to technical difficulties. Of the 17 interviews, 16 were conducted in Swedish and one in English. No financial compensation was provided other than reimbursement for travel expenses if requested.

One of the interviewers was the first author (woman), who is a PhD student and medical doctor without prior qualitative research experience. The second interviewer was the second author (man) with previous experience conducting qualitative in-depths interviews. Informants were given the opportunity to state a preference between being interviewed by a man or a woman given the sensitive subject matter. Both interviewers followed the same interview guide. Neither work clinically with patients with hypospadias. While the first interviewer is a medical doctor and does research on hypospadias, the second researcher has limited subject matter and no clinical qualifications but extensive experience in sexual and public health education, research, and advocacy. Given the discrepancy in experience, the aims and framework for the study were agreed upon clearly in advance and discussed continuously throughout the study progression. Any assumptions made by the interviewers prior to the study or throughout the process were discussed and contrasted within the research group. Neither interviewer had any form of relationship to any informant they interviewed prior to the interview. However, some of the younger informants had received care from some of the co-authors on this study. All informants were assured that what they shared could in no way negatively affect them or their access to care. Questions asked by the informants during the interview about hypospadias were when possible postponed to the end and referred to specialists in the team, to avoid inappropriate influence by the interviewer’s knowledge or view on the data.

# Interview guide (Translated to English)

This interview guide formed a basis for all the semi-structured in-depth interviews. Each interview was unique, and the different topics were covered in different orders and to varying degrees depending on the informant. Primarily open-ended questions were used. Probes included literal or interpretative summaries, asking the informant to elaborate or clarify, and more direct probes when necessary to increase understanding. Examples of probes are included in the interview guide. A couple of small edits were made to the interview guide throughout the process, but no topics were removed or added. This article only includes a subset of the results about experiences that relate to healthcare.

**Introduction**

*My name is Xx and I’m a researcher at Karolinska Institutet*

*I would like to get a deeper understanding of* ***your*** *experience of living with hypospadias. Sometimes the questions may seem a bit basic but that is because I would like to know how you perceive things and how you describe the terms that you are using. I will record this interview and transcribe it afterwards.*

*To be able to keep this as anonymous as possible, I would like if you did not disclose your name, address or anything which is specific to you. If you say anything by mistake, we will delete it from the transcripts. The transcripts and sound files will be kept securely so that only a few researchers can access them.*

*Before we begin, what word would you like me to use when referring to your penis?*

*I will start the recording now.*

*You have received information about the study and signed an informed consent form. Is that correct? I would like to remind you that you have the right to withdraw your consent at any point.*

**Initial background questions**

*How old are you?*

*Are you currently living in a relationship?*

*Do you have children? If yes, are they your biological children?*

*What is you occupation?*

*How do you define your sexuality?*

*What is your gender identity?*

*To be able to get a deeper understanding of your experiences, I would like to know a bit more about what your *penis* looks like. How would you describe it? [this question is asked when appropriate throughout the interview]*

**THEMES**

*Would you mind first* ***openly describing*** *your experience of living with hypospadias?*

**THEME CHILDHOOD AND PARENTS**

*Could you tell me about the experience of living with hypospadias as a child?*

*Probe: What do you remember yourself and what has been re-told to you?*

*How did your parents relate to your diagnosis?*

*How did your parents talk to you about hypospadias?*

*Which words did they use?*

*How did you perceive your appearance compared to the other boys, for instance after gym class?*

*Probe: Who, situation, feeling. 1 Can you tell me more about it? 2. In which situations did you think of it? 3. Did you have any coping strategies?*

*Can you tell me about others in your surroundings who might have had reactions to the appearance of your penis?*

**THEME HEALTHCARE**

*Can you tell me about your encounters with healthcare?*

*Probe: What do you remember yourself and what has been re-told to you?*

*Probe: Can you describe any hospital visits in relation to a surgical procedure?*

*Which words did the healthcare staff use when you talked about hypospadias?*

*Can you describe either positive or negative impressions of healthcare?*

1. *Can you tell me more about it? 2 How could it have been better?*

*How do you perceive the results of the surgery/surgeries?*

*Probe: Change in perception over time? Thoughts about consequences?*

**THEME SELF-IMAGE**

*How do you feel about or relate to your *penis* today?*

*How do you feel about or relate to your *penis* today in relation to other men?*

*Probe: Can you tell me more about it? In which situation is it an issue? Do you have any coping strategies?*

*Probe: How do you feel about nudity in front of men whom you do not have a relationship to, for instance in a gym or sauna?*

*How would you say you feel now, in general?*

*Probe: In which way does hypospadias, or has hypospadias previously, impacted your general well-being?*

*How do you perceive hypospadias in relation to feeling like a man?*

**THEME RELATIONSHIPS, INTIMACY AND SEX**

*How do you think about hypospadias when starting a new social relationship?*

*How do you think about hypospadias when starting a new romantic or sexual relationship?*

*Probe: 1. How do you address hypospadias with someone you have a sexual relationship with? 2. Can you tell me more about it? 3. In which situations do you think of it? 4. Do you have any coping strategies?*

*Do you experience any sexual consequences which you believe are connected to hypospadias?*

*Probe: If so, in what way?*

**THEME FERTILITY**

***If he does not have children***

*Do you have any thoughts about having children?*

*Leading probe: do you have any thoughts about your ability to have children (or your fertility)?*

***If he has children***

*What thoughts did you have concerning hypospadias in relation to becoming a father?*

**FINAL QUESTIONS**

*Is there anything else you would like to share about your experience of living with hypospadias?*

*Do you mind if we get in touch in the near future if we have any follow up questions?*

*I’m turning off the recording now.*

# Further suggestions for implementation

## Written information

The following is a recommendation of topics to cover in written information for hypospadias patients in adolescence. These recommendations are based on both more specific recommendations from informants, and our overall interpretations of our results.

- Summary of personal medical history, i.e., phenotype and initial anatomy and function, performed surgeries, and other relevant information.
- General information about hypospadias. How common hypospadias is appears to be especially important. This area may also include information about the spectrum of phenotypes, anatomy, brief information about types of surgery, goals, and outcomes of surgery, and possible short- and long-term complications. Illustrations can help make the information clearer. While including photographs in written material sent home to a patient may be sensitive, careful use of photographs in clinical settings with patients and parents may be used to help describe what to expect both pre-, peri- and postoperatively. We also suggest giving an up-to-date and balanced summary of information regarding etiology and long-term outcomes, such as information about the role of testosterone function, fertility, comorbidities etc.
- Wherever possible, provide opportunities for further medical, psychological, or sexological support to adolescents. While this is likely especially important for those with proximal hypospadias, other factors can impact need.
- Information on how, why, and when to contact healthcare in adulthood. If possible, link to online resources that are updated as and when routines change.
- Provide general resources for gathering further information. This may include reading material, websites, or patient-forums. The need for expert medical information or advice, and the need for support from peers or other men with hypospadias, may both exist, and one cannot fully replace the other. We understand that there is generally a lack of reliable resources, and that patient-interest groups often must be initiated and run by patients themselves. Collaborating regionally or nationally to combine funding, expertise, contacts, and time may help to initiate efforts to produce age-appropriate, safe, and clear information, as well as support anyone with hypospadias or parents of children with hypospadias wishing to reach out to the community.

## Specific suggestions from informants

Beyond sharing their own experiences, some informants gave specific recommendations or raised questions which could help impact hypospadias care, research, or policy. These are included here, as they may help inform or inspire readers to look further into some of these areas. Each suggestion may come from one informant or be a summary of suggestions from a few informants. They focus on different age groups and some suggestions are primarily suitable for adult patients.

**Information from healthcare**

1. Inform both about what hypospadias is, but also what is “*normal*” with regards to anatomy and function.
2. Provide specific medical advice and information, for instance:
   1. Hygiene (in general and relating to sexual practices) and decreasing the risk of urinary tract infections.
   2. At an appropriate age be informed of other risks, for instance impaired fertility, hormonal dysfunction, possible cancer risk etc.
3. Mediate contact with someone else with hypospadias to talk to, for instance someone older for younger patients with hypospadias, to help get an idea of experiences, seek support, and discuss more personal concerns.

**Healthcare visits**

1. Help the child develop skills and comfort in discussing intimate and uncomfortable details.
2. Staff should have training in how to discuss and introduce these topics with children.
3. Document the child’s development with regards to not just the physical but also how their communication about hypospadias is developing. What themes have been talked about? What words have been used?
4. Written questionnaires before a visit to introduce intimate and difficult questions.
5. Consider screening high-risk patients with regards to hormonal function and later cancer risk.
6. Help support patients in feeling comfortable and safe during intimate examinations
   1. Give them time to get comfortable or think about whether they want a particular examination, for instance seeing another patient in between.
   2. You might reduce stress in patients by stating that involuntary erections may occur and that it is normal.
   3. Allow the patient to do things themselves under guidance, if they want to, for instance removing catheters.
7. Primarily ask for and consider the needs of the patient around puberty, for instance with regards to psychological support and information, even if the parents do not think there are any issues.
8. Avoid situations where parents can cancel or decline visits for teenage patients without the teenager’s knowledge or consent.
9. Provide direct routes of contact, when possible, with self-referral for adult patients with hypospadias, so they do not have to access specialists via primary care. This can help avoid both unnecessary delays and having to go through unnecessary medical visits with more doctors.

**Surgery**

1. Consider taking photos to document the patient’s anatomy before and after surgery to help follow progress, with consideration for privacy, integrity, and consent.
2. Use and develop more objective and functional tests to help aid surgical decisions for:
   1. Sexual sensitivity, erectile dysfunction, and erection hardness. Are there specific considerations in hypospadias patients?
   2. The impact of penile curvature. More research on what impacts the risk of penile buckling during sexual intercourse and the functional aspects of penile curvature.

**References**

Örtqvist L, Fossum M, Andersson M, et al. Long-term followup of men born with hypospadias: Urological and cosmetic results. *Journal of Urology*. 2015;193(3):975-982.
